# Appeasing Pheromones for the Management of Stress and Aggression during Conservation of Wild Canids: Could the Solution Be Right under Our Nose?

**DOI:** 10.3390/ani11061574

**Published:** 2021-05-27

**Authors:** Pia Riddell, Monique C. J. Paris, Carolynne J. Joonè, Patrick Pageat, Damien B. B. P. Paris

**Affiliations:** 1 Gamete and Embryology (GAME) Laboratory, College of Public Health, Medical and Veterinary Sciences, James Cook University, James Cook Drive, Townsville, QLD 4811, Australia; pia.riddell@my.jcu.edu.au; 2 Institute for Breeding Rare and Endangered African Mammals (IBREAM), 9 Ainslie Place, Edinburgh EH3 6AT SCT, UK; mparis@ibream.org; 3 Centre for Tropical Environmental and Sustainability Science, James Cook University, James Cook Drive, Townsville, QLD 4811, Australia; 4 Mammal Research Institute, Faculty of Natural and Agricultural Sciences, University of Pretoria, Pretoria 0028, South Africa; 5 Discipline of Veterinary Science, College of Public Health, Medical and Veterinary Sciences, James Cook University, Solander Drive, Townsville, QLD 4811, Australia; carolynne.joone@jcu.edu.au; 6 Institut de Recherche en Sémiochemie et Ethologie Appliquée, 84400 Apt, France; p.pageat@group-irsea.com

**Keywords:** wild canid, conservation, metapopulation management, African wild dog, wolf, stress, aggression, immune suppression, reproductive suppression, appeasing pheromone

## Abstract

**Simple Summary:**

Many canid species are declining globally. It is important to conserve these species that often serve as important predators within ecosystems. Continued human expansion and the resulting habitat fragmentation necessitate conservation interventions, such as translocation, artificial pack formation, and captive breeding programs. However, chronic stress often occurs during these actions, and can result in aggression, and the physiological suppression of immunity and reproduction. Limited options are currently available for stress and aggression management in wild canids. Pheromones provide a promising natural alternative for stress management; an appeasing pheromone has been identified for multiple domestic species and may reduce stress and aggression behaviours. Many pheromones are species-specific, and the appeasing pheromone has been found to have slight compositional changes across species. In this review, the benefits of a dog appeasing pheromone and the need to investigate species-specific derivatives to produce more pronounced and beneficial behavioural and physiological modulation in target species as a conservation tool are examined.

**Abstract:**

Thirty-six species of canid exist globally, two are classified as critically endangered, three as endangered, and five as near threatened. Human expansion and the coinciding habitat fragmentation necessitate conservation interventions to mitigate concurrent population deterioration. The current conservation management of wild canids includes animal translocation and artificial pack formation. These actions often cause chronic stress, leading to increased aggression and the suppression of the immune and reproductive systems. Castration and pharmaceutical treatments are currently used to reduce stress and aggression in domestic and captive canids. The undesirable side effects make such treatments inadvisable during conservation management of wild canids. Pheromones are naturally occurring chemical messages that modulate behaviour between conspecifics; as such, they offer a natural alternative for behaviour modification. Animals are able to distinguish between pheromones of closely related species through small compositional differences but are more likely to have greater responses to pheromones from individuals of the same species. Appeasing pheromones have been found to reduce stress- and aggression-related behaviours in domestic species, including dogs. Preliminary evidence suggests that dog appeasing pheromones (DAP) may be effective in wild canids. However, the identification and testing of species-specific derivatives could produce more pronounced and beneficial behavioural and physiological changes in target species. In turn, this could provide a valuable tool to improve the conservation management of many endangered wild canids.

## 1. Introduction

Of the thirty-six species of Canidae, two are critically endangered (Darwin’s fox, *Lycalopex fulvipes*; and red wolf, *Canis rufus*), three are endangered (Ethiopian wolf, *Canis simensis*; African wild dog, *Lycaon pictus*; and dhole, *Cuon alpinus*), and five are near threatened (bush dog, *Speothos venaticus*; maned wolf, *Chrysocyon brachyurus*; sechuran fox, *Lycalopex sechurae*; short-eared dog, *Atelocynus microtis*; and island fox, *Urocyon littoralis*). Many other species of canid are rare with most populations in decline [1]. Increased human expansion is directly correlated with a loss of natural habitat for wildlife [2]. Most species of carnivore, including wild canids, naturally disperse long distances to find mates, prey, and new territory and, as such, are at high risk from anthropogenic pressures [2]. Human pressures include poaching, persecution, road/train accidents, disease transmission from domestic species, and reduced available habitats [3].

When small and/or isolated populations of a naturally dispersing species exist, the level of genetic divergence between each population increases due to increased inbreeding [4]. Reduced immigration and emigration between isolated habitats could rapidly lead to reduced species fitness, as has been demonstrated in canids globally. In wild Scandinavian grey wolves (*Canis lupus*), pup survival through their first winter was reduced where inbreeding was increased [5]. Inbreeding in captive grey wolves negatively affected adult reproduction and juvenile weight as well as the longevity of adult animals [6]. Conversely, merging founding lineages of captive Mexican wolf (*Canis lupus baileyi*) increased fitness traits such as the proportion of live births, litter size, and pup survival [7]. In the African wild dog, Ethiopian wolf, and Mexican wolf, the genetic variation of fitness-related genes in the major histocompatibility complex is low compared to that of other wild canids (Table 1) [8]. Such reductions in alleles are thought to be caused by genetic bottlenecks, which could lead to reduced individual fitness [8,9,10]. 

Human conservation interventions, such as maintenance of sustainable captive and wild populations, aim to safeguard these species from extinction [16]. The risk of inbreeding can be overcome by the translocation of genetically valuable animals between fragmented populations [17]. However, only between 11 and 53% of bird and mammal translocations result in a self-sustaining population [18,19]. A common problem limiting translocation success is animal dispersal away from the release site, which occurs in canids if they are unfamiliar with the area [20]. To reduce dispersal from release areas and facilitate pack cohesion prior to release in social canids, wild-caught animals are often temporarily held in captivity at the release site during the translocation process [20]. Stress has been recognised as a major constraint to the success of translocation in wild canids [21,22]. When translocated animals experience prolonged stress, a number of problems arise, including reduced social cohesion, poor pair-bonding, reduced reproduction, increased rates of infanticide, immune suppression, appetite suppression, and energy mobilisation at the cost of energy storage [23,24,25]. In this review, the consequences of translocation will be discussed in terms of stress and aggression, as well as the current management options and emerging pheromone treatments to mitigate these effects. Moreover, the physiology of pheromone perception, species-specificity, and the mechanism by which appeasing pheromones modulate stress and aggression will be reviewed. 

## 2. Conservation Management of Wild Canids

Metapopulation management, where spatially isolated groups of individual animals are translocated between fragmented habitats [26], has been proposed as a useful method for the recovery planning of threatened canids. This strategy has been recommended for the Ethiopian wolf, red wolf, grey wolf, and African wild dog [6,27,28,29]. The extent of activities in each of these programs may differ depending on goals, release techniques, and environmental aspects (such as predator/prey abundance, size of existing populations, and size of release site [1]). Both successful and unsuccessful reintroduction attempts have been previously documented in grey wolves, red wolves, Mexican wolves, African wild dogs, and the swift fox [27]. Most successful reintroductions are supported by local landowners, and involve holding the animals in captivity prior to release to acclimatise the animals and promote breeding soon after release [27]. Many wild canids have been released and monitored in Europe and America but the ongoing management of populations between fragmented habitats appears limited, in part due to limited funding and population stability [27]. 

Currently the only wild canid actively managed as a metapopulation is the African wild dog, a highly endangered canid that, in the last century, has been limited to just 7% of its historical range [16,30]. This reduction in available habitat has been accompanied by mass persecution and hunting, which has reduced the wild population from an estimated 500,000 to approximately 6600 adult animals [31]. Given that Kruger National Park is the only suitable conservation area large enough to support a self-sustaining population in South Africa, a metapopulation approach has been adopted to manage other fragmented habitats [29,32]. This is achieved by managing a series of small, isolated sub-populations as a single population through the movement of individuals between suitable areas [32]. This approach involves the translocation and artificial pack formation of animals between habitats, thereby mimicking natural immigration and emigration patterns [32]. Today, managed wild populations of African wild dogs have shown greater pup and yearling survival and annual population growth than is recorded in established and unmanaged populations [33]. As one of the few wild canids that are actively managed, the African wild dog presents a unique opportunity to review the effect of such conservation intervention on behaviour and physiology.

### 2.1. Translocation of Wild Animals

Translocation is the deliberate and mediated movement of wild individuals from one part of their range to another [34]. This technique is increasingly used to augment populations that are in decline or to restore extirpated populations of animals [35]. Translocations aim to create self-sustaining populations, and the ability of the animal to survive through the establishment phase strongly affects the outcome [17]. Translocation objectives should include (i) animal survival post release; (ii) animal settlement in the release location; and (iii) reproduction of the released animals in the area of translocation [36,37,38]. 

Historically, there have been a number of attempts to translocate both captive-bred and wild-caught canids. In the United States, most attempts to supplement existing grey wolf populations with wild-caught animals throughout the 1970s were unsuccessful due to human persecution and other anthropogenic factors [39,40]. The red wolf was reintroduced into North Carolina through translocation from 1987 to 1995, resulting in a dispersal of the wolf over large areas and the successful establishment of a thriving population [41]. In Canada, the swift fox was reintroduced using captive-bred and wild-caught animals, with mixed results [42]. The initial African wild dog translocation attempts were unsuccessful, largely due to the release of captive-bred animals that lacked the necessary survival skills in the wild [43]. More recently, attempts to release captive-bred with wild-caught individuals have resulted in greater success rates [43,44].

### 2.2. Artificial Pack Formation

Artificial pack formation involves the temporary capture of dispersing animals and their subsequent joining with unfamiliar conspecifics of the opposite sex in order to form a reproductive unit [45]. Such pack formations can be used to either supplement populations or to re-establish a species within a habitat [46]. Artificial pack formation increases the genetic diversity of isolated populations, ensuring continued species fitness [47]. Releasing canids as pre-formed packs increases translocation success, with animals more likely to reproduce and form territories within the release site, thus increasing survival post release [48]. Temporarily holding grey or red wolves in family groups or pairs prior to their release into novel environments results in greater pack stability and retention at the release site than for the wolves released as individuals [20,49,50]. It is important to consider integration strategies when introducing unfamiliar animals to each other in order to facilitate the formation of new social groups prior to release [51].

The technique of holding animals in temporary enclosures (bomas) prior to release has proven useful in a range of carnivores, ensuring that the animals remain within the release site [18,52,53,54]. In Norway, Artic foxes (*Vulpes lagopus*) are released as litters at eight months of age, or as opposite-sex pairs, from captive breeding programs following habituation in natural enclosures. This approach has resulted in the successful re-establishment of populations in the Alpine region of Norway [22]. It has been observed that African wild dogs released together with the aim of pack formation regularly disperse away from one another unless social cohesion occurs prior to release [16]. Young African wild dogs naturally disperse away from their natal packs as single-sex groups during their first year of sexual maturity [33,55], and search for groups of unrelated animals of the opposite sex with which to form a new pack [21]. Initial contact between unfamiliar dispersing groups is often marked by aggression due to a lack of established dominance hierarchies [56]. These interactions will either result in pack formation or continued dispersal. Holding unfamiliar males and females in a boma allows them to test their compatibility with each other, resulting in either the formation of an established pack or a pack annulment [57]. When opposite-sex groups successfully form packs, separate male and female dominance hierarchies are formed. The alpha pair have almost exclusive mating rights, and their subordinates help to raise young, guard territory, and hunt [56,58,59]. 

Artificial pack formation is often performed in conjunction with translocation, with the aim of mimicking their natural dispersal pattern, and forming socially cohesive packs that will eventually lead to functional breeding groups [58]. However, one of the largest limiting factors to the success of artificial pack formation and translocation is the stress caused by captivity and the related aggressive behaviours that can result in injuries and mortalities [60,61]. During translocation, chronic stress results from the disruption of hierarchical or familial bonds, temporary pack separation, and periods of captivity involving human husbandry, causing social instability [62,63,64]. Moreover, many social species often experience stress during the establishment and maintenance of social hierarchies due to the increased dominance behaviours, such as fighting and aggression [65,66]. The boma design, the size of the groups brought together, and the way in which animals are brought together should be designed to minimise the length of time it takes for cohesive packs to form, while also providing all the basic necessities to animals.

Boma construction can include either single or split compartments depending on the financial and human resources available [21]. The type of boma (one- or two-compartment) and the length of time spent within it varies greatly at different translocation sites, though the management of animals is performed so as to facilitate rapid social cohesion [21]. The amount of time spent within a boma may depend on the group size, with larger groups requiring more time to form a stable pack regardless of the boma’s design [21,67]. Conversely, smaller groups brought together appear to socially integrate more rapidly in single compartment bomas [21]. Odour familiarity has been found to decrease the aggression related to social integration [61], and it is now standard practice to rub wild-caught African wild dogs against each other while they are immobilised [61]. The sex ratio can also impact social integration, with a higher proportion of females to males resulting in a more rapid pack formation [21]. When social integration occurs and a cohesive pack forms between opposite-sex animals, affiliative behaviours can be observed, including resting in close proximity [57,67] and reduced long distance calls [67]. However, all forms of captivity result in higher levels of stress hormones in African wild dogs, and this is increased in wild-caught animals compared to captive-bred individuals [60].

## 3. Stress and Aggression during Conservation Management

When a stressful object or event is perceived/experienced, it results in a stress response that causes physiological and behavioural changes [23,35]. The magnitude and length of these changes is controlled by the hypothalamic-pituitary-adrenal (H-P-A) axis [23]. This pathway regulates the release of glucocorticoids such as cortisol and/or corticosterone [68]. The consequences of glucocorticoid release include increased energy mobilisation at the cost of energy storage, and the suppression of growth, reproduction, immunity, and the inflammatory response [23]. Episodes of stress and aggression can either be acute (lasting minutes to hours) or chronic (lasting days to months) [69]. During acute stress, these adaptations are beneficial with a diversion of physiological and behavioural processes to immediate survival [70]. However, prolonged activation of the H-P-A axis during chronic stress results in the detrimental suppression of physiological processes and subsequent behavioural coping, such as displays of heightened aggression [71,72]. 

Increased aggression is often correlated with an increase in the androgen hormone testosterone and the hormones dehydroepiandrosterone (DHEA) and androstenedione [73]. Testosterone is produced under the influence of luteinising hormone, the level of which is controlled by the hypothalamic-pituitary-gonadal (H-P-G) axis. Upregulation of the H-P-G axis is associated with elevated testosterone levels, which has been linked to both male and female aggression, especially during the breeding season in social species [66,74,75]. Prolonged elevated levels of testosterone and cortisol are directly correlated with behaviours such as increased aggression and reduced levels of parental care/bonding, which negatively impact both male and female reproductive success, as well as dysregulation of immune function in a number of different species [76,77,78,79].

The conservation management of wildlife often requires the capture, handling, and temporary housing of animals in captivity, all of which may induce stress [35]. For example, a delayed release during the translocation of eastern bettongs resulted in increased faecal glucocorticoid metabolite (fGCM) concentrations [80]. It is thought that translocation of swift foxes caused chronic stress, which negatively impacted their post-release survival [81]. Moreover, in one study, stress was thought to be responsible for driving self-inflicted physical injury during a translocation of grey wolves [20]. Despite this, there is limited monitoring of the parameters of physiological stress described in the literature during the conservation management of wild canids, even though behavioural distress is commonly reported [20,22,81].

Interestingly, captive African wild dogs show significantly higher stress-related fGCMs than their free-living counterparts [82]. Similarly, permanent and temporary captivity results in similar fGCM concentrations, both of which are higher than in free-living African wild dogs, although this study relied on a single defecated sample, and did not report the length of time the animals had been kept in temporary captivity prior to the sample’s collection [60].

In the African wild dog, increased glucocorticoid concentrations in the blood are directly correlated with increased aggression [83]. During the breeding season, male African wild dogs experience higher glucocorticoid concentrations that are concurrent with higher testosterone levels, with dominant males having higher levels of both hormones than their subordinates. [56,62,84,85]. The level of aggression displayed by dominant animals is directly related to the attainment and maintenance of dominance hierarchies, with alpha animals having elevated glucocorticoids year-round compared to their subordinate pack mates [82,86]. Furthermore, dominant animals are more aggressive and fight more than their subordinates during periods of mating and, as such, the breeding season is a suboptimal time for translocation and artificial pack formation [56]. This, in part, is due to the observation that high levels of aggression after group integration can lead to mortalities during artificial pack formation [67].

### 3.1. Impact on Reproduction

Translocation stress induced by suboptimal conditions during temporary captivity in the Arctic fox was thought to cause a failure of females to enter oestrus during the first and second breeding season after release [22]. In ex situ-bred red wolves, fGCM levels can directly influence the frequency or propensity to engage in breeding behaviour, with females observed engaging in copulatory ties having low baseline fGCM levels [87]. Interestingly, fGCM levels are higher in dominant grey, Iberian (*Canis lupus signatus*), and Ethiopian wolves as well as African wild dogs compared to their subordinates, but this does not appear to reduce a dominant pair’s reproductive potential [75,88,89,90]. Furthermore, semen quality in African wild dogs does not appear to differ between dominant and subdominant males [75]. Despite a lack of evidence to suggest that increased fGCM levels, as a result of stress, physiologically inhibit reproductive potential in canids, elevated fGCM levels could influence parental care and reproductive behaviours, such as mate-guarding, with aggressive behaviours leading to reduced litter success [56,75,88,89,90]. It is thought that prolonged confinement and the artificial pack formation of canids may result in chronic stress to such an extent that reproductive success post-release could be compromised [21]. Captive African wild dogs have relatively high rates of pup mortality, despite improved husbandry and veterinary care [91]. According to the North American Studbook, the mortality of entire litters and individual pups was 52 and 53%, respectively, between 1993 and 2013 [92]. In South African captive breeding programs, individual pup mortality is as high as 63% [93]. The factors that may contribute to such a high mortality rate include the age of the female at first breeding, the number of previous litters the female has had, and the level of inbreeding between captive packs [91,94]. Similarly, in the red wolf, primiparous and multiparous females have greater reproductive success and lower baseline fGCM levels than nulliparous females [87]. Furthermore, litter success is relatively low, with only 20% of captive females producing litters in 2016, compared to 31% among in situ populations [87]. As mentioned previously, female African wild dogs held in permanent captivity experience significantly more stress than free-living counterparts [60,82]. This can be attributed to their limited ability to exhibit natural behaviours, which may destabilise the social structure, co-operation, and cohesion within the pack, resulting in reduced reproductive success [60].

Both acute and chronic stress can reduce the levels of parental care in non-canid species. In non-human primates, stressors (including a lack of social support, crowding, and being the recipient of aggression) can increase the rate of infant abuse [95,96,97,98]. Human mothers that experience depression engage more often in negative and disengaging behaviours towards their children and have lower rates of positive behaviours compared to non-depressed mothers [99,100]. Abusive parents often have an overly sensitive stress response to infant distress [101]. In males, concurrent high levels of cortisol and testosterone are negatively associated with the quality of caregiving [102]. Thus, high levels of stress may also be associated with reduced parental care in wild canids; however, more research is needed to determine whether a correlation exists in any species of canid. Despite the apparent stability of African wild dog packs, the abandonment of pups from first litters does occur, especially when pack hierarchies remain unstable [67], or when mothers have limited experience [91]. The monitoring of packs after release is often limited due to practical constraints and, as such, hierarchy instability and litter abandonment may be under-reported and may also contribute to pup mortalities in artificially formed packs.

### 3.2. Impact on Immune Function

Vulnerability to disease is increased by stress-related immunosuppression in animals [103,104]. Chronic stressors cause a decrease in almost all functional immune measures as a result of chronically high levels of cortisol [105]. This increases an individual’s susceptibility to infection [106,107], delays wound healing [108], and impairs the immune response to vaccination [109,110,111]. The degree to which cortisol suppresses the immune system may vary depending on the duration of the stressor [105]. During acute stress, brief natural stressors elicit a short-lived stress response and thus immune suppression is short-lived. When chronic stress is experienced, the inability to predict when a stressor will end, such as during translocation and captivity, leads to long term immune suppression [105]. In cases of chronic aggression, the immune system may become maladaptive [112]. A prolonged elevation of androgens can compromise immune function [113] and is associated with delayed wound healing, the dysregulation of cytokines at wound sites, and heightened pro-inflammatory cytokines and other immune cells [112,114,115,116].

Chronic aggression and stress leading to immune system dysregulation and suppression during the translocation of canids could impact the ability of animals to cope with pathogen threats in the habitat they are released into. A number of pathogens are of concern globally to canids, such as canine parvovirus, canine distemper virus, and rabies virus. Canine parvovirus (CPV) has been identified in grey wolves and coyotes in Yellowstone National Park, though seroprevalence suggests that disease-induced mortality is low, even in young animals [117]. Canine parvovirus has limited risks for adult African wild dogs but can result in a 38–40% reduction in litter size and pup survival during outbreaks [65,118]. Canine distemper virus (CDV) has caused high pack mortalities and population eradications in African wild dogs [65,119,120], though some adults have been found with antibodies to this disease [65,121]. Similarly, CDV outbreaks in Ethiopian wolf populations typically caused mortality in sub-adults and juveniles [122]. In Yellowstone National Park, CDV outbreaks affected both grey wolf and coyote populations, particularly pups [117,123]. Lastly, rabies virus outbreaks have caused high rates of pack mortality and population eradication in African wild dogs [124,125,126]. In Ethiopian wolves, rabies caused the mortality of over 70% of packs and has previously been the cause of local population extinctions [127,128,129,130,131]. The risk of infection, outbreak, and mortality resulting from pathogens of concern could be exacerbated during translocations and artificial pack formations due to the immune suppression arising from chronic stress.

It has previously been proposed that the stress of immobilisation, radio collaring, and, in particular, vaccination could compromise the immune system of African wild dogs. This erroneous belief stems from reports of mortalities and animal disappearances shortly after the vaccination of African wild dogs [125,132,133,134,135]. These disease outbreaks occurred prior to the onset of vaccine immunity and unmanipulated packs also suffered pathogen-related mortality during these outbreaks [132]. More research is required to determine how stress impacts the immune response of wild canids during conservation interventions.

## 4. Management of Stress and Aggression

Given the serious consequences of chronic stress and aggression in canids during conservation activities, it is important to consider the ways in which these can be alleviated. In domestic dogs, castration is a common tool to reduce aggressive behaviour [136]. Gonadectomy in aggressive male and female dogs resulted in 61 and 53% of dogs becoming gentler, respectively [137]. Permanent (gonadectomy) and reversible (GnRH vaccination) castration has been used in African wild dogs housed in European zoos with debatable effectiveness (Richard Barnes, Personal communication). However, the use of castration to ablate aggressive behaviour in endangered wildlife is counterintuitive when often the goal is to breed as many of these genetically valuable animals as possible. Other methods of behavioural control may be more nuanced and efficacious. 

### 4.1. Pharmaceuticals

In domestic dogs, pharmaceutical treatments for anxiety include the administration of a range of different psychoactive drugs, such as fluoxetine (a selective serotonin reuptake inhibitor [138]), tricyclic antidepressants, benzodiazepines (a nervous system depressant), and buspirone (an azapirone neuroleptic) [139]. Of these drugs, fluoxetine is the most commonly prescribed due to its proven efficacy for treating anxiety and aggression [139]. These medications are prescribed to reduce the behavioural implications of stress due to separation anxiety, a phobia of thunderstorms, fear, and hospitalisation [140,141]. However, these drugs can have undesirable side effects, such as diarrhoea, vomiting, sedation, hypotension, agitation, ataxia, and excitement [139].

The use of pharmaceuticals to manage stress and aggression during wildlife management is controversial. The administration of midazolam and azaperone to wild-caught mule deer did not reduce physiological (heart rate, blood oxygen saturation, body temperature), hormonal (fGCM, serum cortisol) or behavioural (vocalisations, kicking) stress [142]. Furthermore, observations of hesitation, stumbling, and falling in mildly tranquilized deer after their release from a drug-induced lethargy could compromise animal welfare [142]. Fluoxetine has been reportedly administered to Asiatic bears (*Ursus thiberanus*) [143], brown bears (*Ursus arctos*) [144,145], polar bears (*Ursus maritimus*) [146], and Bengal tigers (*Panthera tigris tigris*) [147] housed in zoos to treat anxiety and pacing behaviours. Reports indicate that during drug administration, pacing behaviours are reduced and the amount of time spent resting is commonly increased, which is a possible sign of drug-induced lethargy [142,144,146]. Administration of fluoxetine to control aggression and excessive mounting behaviours in castrated male red-necked wallabies (*Macropus rufogrisues*) resulted in mild sedation for several days after administration and was effective at reducing aggression only when administered daily for a number of months [148]. European zoological institutions have used fluoxetine hydrochloride and sedative drugs during the introduction and translocation of African wild dogs [63]. Such treatments did not appear to reduce stress in most situations [63] and their sedative effects could be detrimental to the social interactions between pack mates, thereby destabilising established hierarchies. In domestic dogs, a positive relationship was found to exist between cortisol and DHEA pre- and post-fluoxetine treatment. However, aggressive dogs’ serum cortisol, DHEA, and serotonin remained higher than control animals post-treatment, and dogs that had high serum serotonin concentrations pre-treatment showed poorer improvement in behaviours, cortisol, DHEA, and serum serotonin than control dogs [149]. In rats, fluoxetine has other side effects, including low libido, and delayed or inhibited ejaculation [150]. In summary, drug-induced side effects, such as sedation, reduced reproductive potential, and change in mounting behaviours, could alter dominance hierarchies and social cohesion in wild canids during conservation interventions, making pharmaceutical options for stress management inadvisable.

### 4.2. Pheromones

Pheromones are naturally occurring chemicals that are released by an organism to modulate the behaviour and/or physiology of conspecifics [151]. They offer a novel, natural alternative for the management of animal behaviours. Pheromones are able to regulate endocrine status, signal individual identity, and evoke sexual, nurturing, and aggressive behaviours [152,153]. In mammals, pheromones are excreted from almost all glands and are detected by the olfactory system [153]. They are highly specific to chemoreceptors and a single olfactory glomerulus is activated by each individual chemical component of the pheromone, causing the excitation of interneurons in a precise combination to initiate a particular behavioural response [152]. 

**Figure 1:**
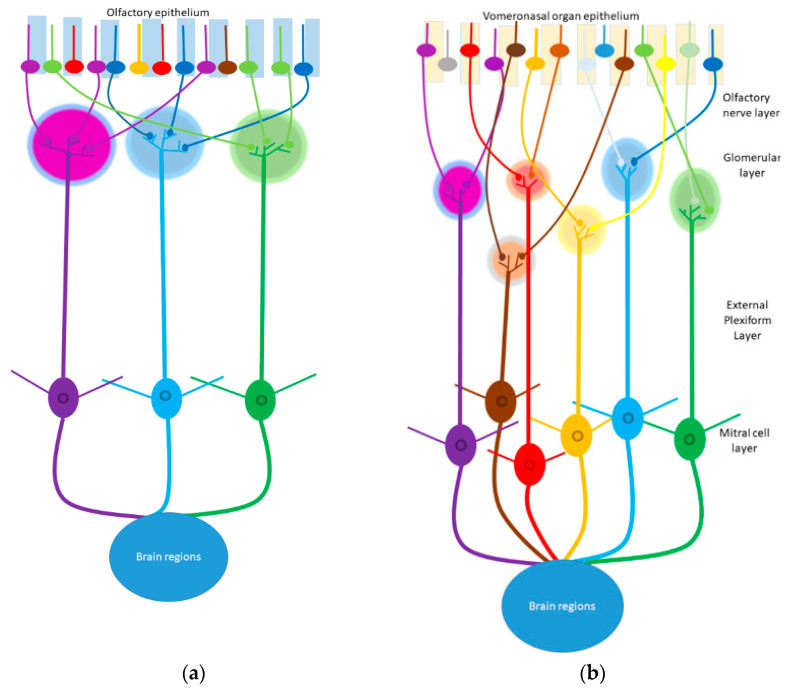
Schematic representation of sensory projections in the (**a**) main olfactory epithelium (MOE) and (**b**) vomeronasal organ (VNO). (**a**) Within the MOE, olfactory sensory neurons in the olfactory epithelium expressing the same specific odorant receptor have axons that innervate to the same glomerulus (represented by the different colours green, blue, and purple), which in turn excite specific mitral cells to act on specific brain regions. (**b**) Within the VNO, sensory neurons expressing the similar vomeronasal receptor innervate multiple small glomeruli, which excite mitral cells and thus specific brain regions. (Each colour represents a population of vomeronasal sensory neurons, that express one different type of vomeronasal receptor. Adapted from [161].

While the mechanism by which pheromones are perceived in mammals is not completely understood, it is thought that the main olfactory and vomeronasal systems, along with additional olfactory organs, are involved in pheromone detection [153,154]. These systems possess a similar histological organisation, with primary sensory neurons that project axons to the mitral cells (second-order neurons) contained in specific regions in the main olfactory bulb (MOB) or the accessory olfactory bulb [153,154]. Primary sensory neurons of the main olfactory epithelium (MOE) are responsible for transduction of certain pheromones to the mitral cells of the MOB [153,155,156]. In the MOB, the mitral cells project to multiple higher centres in the brain, including the amygdala and the piriform cortex. The MOB is the primary target region for olfactory neurons [153,154]. The laminar organisation of this bulb is comprised of a superficial nerve layer with axonal projections of chemosensory neurons. A first-order synaptic region exists between the sensory neurons and the mitral cells, represented by a glomerular layer. The soma of mitral/tufted and granule cells resides in the external and internal plexiform layer [153,154,157]. Within the MOB, glomeruli are anatomically separated and encapsulated by peri-glomerular cells [153,154]. Axons of olfactory sensory neurons express a particular odorant receptor that converges on only two glomeruli in the MOB [153,154,158,159,160]. Mitral/tufted cells within the MOB bear only one apical dendrite that extends to certain brain regions; thus, they transmit signals from glomeruli to pyramid neurons in the olfactory cortex, bypassing thalamic relay (Figure 1) [153,154,157].

**Figure 2:**
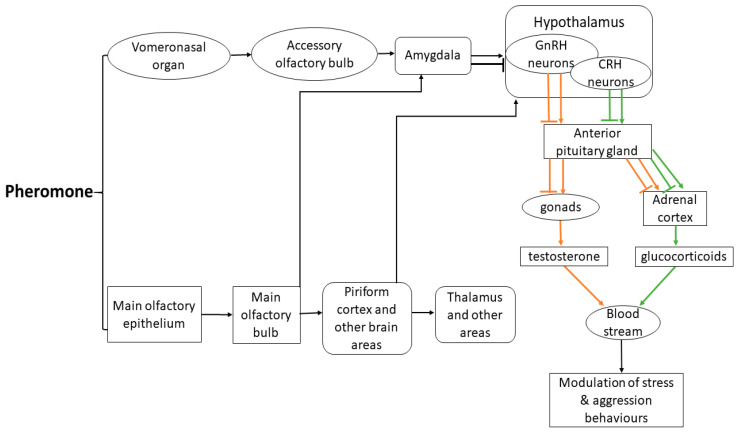
Pheromone perception pathways from the main olfactory epithelium and vomeronasal organ, through the main and accessory olfactory systems, and thalamic and hypothalamic higher brain regions, ultimately leading to a hormonal and/or behavioural response. Stress pathway upregulation (green ↓) or suppression (green ⊥); aggression pathway upregulation (orange ↓) or suppression (orange ⊥). Adapted from [154].

Unlike the separation between the MOE and the MOB, the vomeronasal epithelium is part of the vomeronasal organ (VNO) that is recessed, with detection relying on pheromone molecules being dissolved in nasal mucus and being sucked into the lumen of the organ [153,154,162]. Access of stimuli to the lumen is modulated by the vasodilation/constriction of blood vessels and sinuses lateral to the lumen [163,164,165,166], which is under hormonal control and can be actively modulated by some pheromones [167]. Vomeronasal sensory neurons are found in a pseudostratified epithelium in the basal and apical aspects of the vomeronasal organ [153]. Pheromones cause membrane depolarisation and increase the action potential firing rate of sensory neurons in the VNO [168,169]; however, the signal transduction cascade is largely unknown. Signals from the VNO sensory neurons excite a given vomeronasal receptor and send axonal projections towards the anterior and posterior accessory olfactory bulb. Neurons that excite the same receptor have axons that merge into 10–30 glomeruli [154]. Differentially, inputs from neurons expressing different receptor types are received by one glomerulus [153,154]. Mitral cells of the accessory olfactory bulb connect to the medial amygdala, posteromedial cortical amygdala, the accessory olfactory tract, and the bed nucleus of the stria terminalis [154,170]. Ultimately both the MOE and VNO pathway projections lead to specific areas of the amygdala and the hypothalamus modifying the behaviour and endocrinology of animals (Figure 2).

The effect pheromones have on neuroendocrine status are mediated by the hypothalamus [171]. Pheromones that act on the H-P-A axis generate action potentials along paraventricular neurons of the hypothalamus, and repeated exposure to stress causes an increased expression of receptors that act to release corticotrophin releasing hormone (CRH) [154]. Activation of the H-P-A axis by pheromones causes the synthesis and release of vasopressin and CRH, which act via the pituitary and adrenal glands to trigger a cortisol response in the presence of a stressor (Figure 2) [154]. Pheromones that activate the H-P-G axis activate gonadotropin releasing hormone (GnRH) neurons, which lead to the stimulation of luteinising hormone and follicle stimulating hormone that stimulate the development and function of the gonads and the output of testosterone and other androgens that modulate aggression (Figure 2) [66,74,75,172]. Importantly, pheromones can also downregulate the H-P-A and H-P-G axes by reducing paraventricular nucleus receptor expression and limiting the anterior pituitary gland release of luteinising hormone and follicle stimulating hormone, thereby reducing stress and aggression [154,173]. The pheromones that act on GnRH neurons are believed to control endocrine responses in the hypothalamus [154,174,175]. Studies using genetic approaches have concluded that the GnRH neurons in the hypothalamus are synaptically connected to thousands of neurons in over 50 diverse brain areas, including the main olfactory regions [176,177]. This suggests that GnRH neurons may influence a large variety of brain functions and, thus, the neuroendocrine status [154].

Structurally, pheromones can be made up of small volatile molecules, steroid derivatives, peptides, or large protein-ligand complexes [153]. Pheromone-containing secretions, such as urine, sweat, saliva, and tears, contain natural product blends that can be vastly complex [153]. Pheromones are chemically diverse, although closely-related species often emit pheromones that are structurally similar [178]. Despite this, different ratios and components that constitute the same pheromone in different species contribute towards its species-specificity, preventing cross-reaction [179]. In many fish species, a preference for water that previously held conspecifics compared to heterospecific odours provides strong evidence that conspecific odours rather than visual cues are used to mediate species recognition [180]. In sea snakes, female lipids excreted from the skin create chemical cues that control male courtship behaviour, and the species-specific nature of such pheromones within these lipids prevents hybridisation by discouraging courtship behaviours between closely-related species [181]. Social insects are able to distinguish between known and unknown conspecifics due to differences in the pheromone and individual odour profiles [152]. In mammals, chemical signals are thought to be unique to each species due to the high level of potential interference from the odours of other species within the environment [182]. Vomeronasal type-1 receptors (V1R) are partly responsible for pheromone detection in mammals and are responsible for mediating species-specific responses. The V1R in carnivores has between 50–90% similarity with domestic cats, cows, and humans, which may help to explain how closely related species may distinguish the pheromones of conspecifics and heterospecifics [183]. Despite this, pheromones that have conserved chemical signatures across species can generate a response in the olfactory receptors or even processing in the central nervous system of closely related species [182]. However, differences in genes of the vomeronasal organ and main olfactory epithelium have been found between species and subspecies, which may cause pheromones of closely-related species to be less effective [182,184,185,186].

## 5. Pheromones for Conservation

Research on the pheromones of large carnivores is limited and to date has focused on territory-related pheromones in urine and faecal marks [187,188,189]. Pheromones that mark territory could be used as spatial signalling compounds or ‘bio-boundary markers’ to prevent dispersal into agricultural land where large carnivores may face persecution [189]. The targeted exposure of African wild dogs to species-specific scent marks from foreign individuals resulted in packs moving away from the marks and towards the centre of their defined home range [190]. While the detection of volatile components of biological secretions has become more common [187,188,189,191], there is a lack of research on the behavioural and physiological changes triggered by these pheromone messages. Among the limited studies, most of the research has focused on the effect of domestic animal pheromones on behaviour and/or physiology in captive wildlife [63,192,193]. Preliminary data suggest that pheromones could be useful to control or alleviate undesirable behaviours in wild canids.

### 5.1. Appeasing Pheromones

Appeasing pheromones are chemical messages that are commonly released by lactating females, which act biologically to calm and reassure newborn animals [173,194]. Species-specific appeasing pheromones have been identified and isolated from a number of different domestic species [173] and have been reported to reduce aggression and fighting in cats, dogs, pigs, and horses [194,195,196,197,198]. Furthermore, in pigs, this pheromone may increase feed intake as well as reduce fighting related lesions [197]. In dogs, it reduces signs of stress, such as whale eyes, licking, yawning, vocalisation, and fearful postures [194].

The appeasing pheromone can be identified according to a conserved appeasing ratio of oleic, palmitic, and linoleic acids. In addition to this core appeasing message, species-specific components begin with myristic acid and are composed of other fatty acids with variable ratios across species. A basic composition of oleic acid, palmitic acid, linoleic acid, and derivatives thereof can have an appeasing effect in all mammals; however, preferred embodiments with enhancer pheromone composition for different species have been identified (Table 2) [199]. These enhancer compositions are species-specific in mammals―they act to enhance or act synergistically with the core pheromone message and increase their behavioural and physiological effectiveness in a target species [199].

### 5.2. Dog Appeasing Pheromone

The commercially-available dog appeasing pheromone (ADAPTIL^®^, previously DAP; Ceva Sante Animale, Libourne, France) has been reported to reduce behaviours associated with fear and anxiety in puppies entering new environments [200] and in adult dogs during transport, veterinary settings, thunderstorms, and rescue shelters [173,194,201,202,203]. Treatment with ADAPTIL^®^ has been found to be as effective as pharmaceutical treatments for the reduction in stress-related behaviours that occur as a result of separation anxiety [204]. This pheromone can be administered as an imbibed collar, diffuser, or aerolite spray. A blinded, placebo-controlled study of the effect of ADAPTIL^®^ on stress reduction in dogs subjected to a thunderstorm simulation found that while all dogs showed stress behaviours above the baseline, the fear response between thunder noises was reduced in dogs with ADAPTIL^®^ collars compared to placebo collars [202]. Imbibed collars have been found to reduce anxiety-related aggression more effectively than diffusers, although the duration of exposure may affect these results since collar studies often exposed animals to ADAPTIL^®^ for 24 h prior to the behaviour analysis [194,205]. In contrast, when dogs were only exposed to diffused ADAPTIL^®^ in a veterinary setting 7 min prior to examination, no reduction in stress and aggression behaviours occurred, although their duration was reduced, and the frequency of relaxed states was increased [194]. In a blinded, placebo-controlled dog trial, ADAPTIL^®^ released from diffusers for 2 days prior to parturition and for 3 weeks post-partum in maternity kennels increased the amount of time mothers nursed in a laying position and spent in close contact with their pups [206]. Despite promising behavioural results, most studies rely on subjective observation, coupled with behavioural measures that can be less sensitive than physiological measures of stress [194,207]. While it is possible to infer emotional states from indirect measures, such as behaviour, any given state can be expressed by a variety of behaviours [194]. Additionally, much of the literature on the use of ADAPTIL^®^ lacks baseline measures for individual animals (with some work relying entirely on owner surveys) or lack a blinded experimental design that would mitigate the introduction of observer bias [201,204].

Limited reports have investigated the underlying physiological mechanisms affected by appeasing pheromones, but preliminary research suggests that, with the exception of one study [63], synthetic appeasing pheromones do not appear to affect the H-P-A axis to reduce stress, even if they are species-specific [192,208,209]. These studies used a pheromone spray application method that, as stated previously, may not be as effective if not continuously released for extended periods of time. However, recent cross-species research suggests that the appeasing pheromone may work via the H-P-G axis at the level of testosterone, thereby reducing aggression and coping behaviours during stress [192]. Nevertheless, based on preliminary data collected by Vlamings [63], it remains to be determined whether species-specific appeasing pheromones may be more effective in suppressing the H-P-A pathway. Thus, collectively, species-specific appeasing pheromones could be a useful tool to modify both the stress and aggression pathways in wild canids during conservation interventions.

### 5.3. Application of Dog Appeasing Pheromones to Wild Canids

Currently, the African wild dog is the only wild canid in which appeasing pheromones have been tested, and an initial administration of dog appeasing pheromone to this species has shown some promise. Captive African wild dogs appear to perceive ADAPTIL^®^ and exhibit a higher rate of favourable behavioural responses in the treated area, such as resting, sniffing, urinating, licking mandibular, and panting with tongue out of mouth during perception studies [63]. To further determine the perception and behaviour modification due to the ADAPTIL^®^ treatment, three packs that were displaying aggression between animals were separated, treated with ADAPTIL^®^ collars and spray, and then reintroduced. On the day of reintroduction, all of the packs showed dominant and affiliative behaviours, as expected, but two packs also exhibited severe and ritualised aggression. In Pack One, both dominant and affiliative behaviours persisted with no change in their frequency over the study period. In Pack Two, the frequency of dominant behaviours declined significantly over time without a change in the frequency of affiliative behaviours. In Pack Three, which exhibited no severe aggressive behaviours until day nine after reintroduction, the frequency of affiliative behaviour declined significantly over time [63]. Unfortunately, the limited sample sizes, lack of control groups, high variability between packs, and lack of initial baseline data make it difficult to conclude from this study whether ADAPTIL^®^ was able to ameliorate aggression. In a third experiment, three female African wild dogs were separated from their respective packs, housed in isolation, and treated with ADAPTIL^®^ collars to investigate the effect of ADAPTIL^®^ on physiological stress levels. In these females, the mean fGCM concentrations were reduced by 0, 68, and 82%, respectively, compared to pre-treatment levels, suggesting that ADAPTIL^®^ may help to suppress the H-P-A axis [63]. 

A follow-up double-blinded placebo-controlled study found that, compared to the controls, well-established zoo-based African wild dog packs treated with ADAPTIL^®^ exhibited reduced contact dominance and active submission behaviours after separation, immobilisation, and reintroduction events as part of a routine health assessment in captivity [192]. Administration of a spot-on ADAPTIL^®^ solution (as two 5-milliliter aliquots between the shoulder blades and at the base of the tail) to male African wild dogs during this medical intervention did not significantly reduce the fGCM concentrations after reintroduction [192]. However, compared to placebo-treated animals, the ADAPTIL^®^ treatment was found to suppress the faecal androgen metabolite surge that normally occurs after reintroduction in response to acute stress. This was accompanied by a significant shift from contact dominance to non-contact dominance behaviours [192].

It is worth noting the difference, not only in the ADAPTIL^®^ administration, but also in the experimental design in these studies. Vlamings [63] housed each of three females individually for the duration of one study to investigate the effect of ADAPTIL^®^ on physiological stress, while in a separate experiment, the effect of ADAPTIL^®^ on behaviour was investigated in unstable packs that had been previously separated due to high levels of aggression between individuals. In contrast, Van den Berghe et al. [192] measured both behaviour and physiology in the same groups of animals housed in established packs that showed low levels of aggression throughout the study. While both studies used a pre-treatment period to compare the hormone levels before/after ADAPTIL^®^ treatment in the same individuals, control animals were absent in Vlamings (2011) study, while double-blinded placebo-treated control packs were used by Van den Berghe et al. [192]. Collectively, these studies suggest that cross-species ADAPTIL^®^ may suppress the H-P-G axis to control testosterone surges and associated aggression in African wild dogs, but this pheromone appears to have a variably suppressive effect on the H-P-A axis, cortisol, and stress. However, a stronger combined effect on both the H-P-A and H-P-G axes may be achieved if species-specific appeasing pheromones are isolated and administered to each canid species.

Appeasing pheromones could therefore be a useful tool to reduce agonistic interactions in African wild dogs and other wild canids during translocation and the artificial formation of bonded pairs/packs among unfamiliar animals [62,63,64]. The initial extraction and identification of appeasing pheromones has been derived from mammary gland secretions [173,199]. However, they have subsequently also been found in other bodily secretions of the skin and in faeces, which can be collected with greater ease from wild canids [197,210]. Gas chromatography coupled with mass spectrometry is used to quantify the different volatile substances and fatty acid compositions contained within secretions for the presence of the conserved appeasing message of oleic, palmitic, and linoleic acid, followed by the species-specific components [189,199]. Candidate pheromones can then be synthetically replicated using relatively cost-effective, mass-produced chemical components, allowing for the replication of species-specific pheromones that are readily available and feasibly deployed [199]. Bioassays that evaluate behavioural, hormonal, immunological, or neurophysiological responses in a species can then be used to validate the effect of pheromones and their potential benefits [188]. In wild canids, we advise measuring behaviours (e.g., aggression, resting proximity, and submission) and hormones (cortisol and testosterone) that are indicative of stress and aggression pre- and post-pheromone exposure. Their potential benefit to improve reproduction could also be measured through behavioural analysis, breeding outcomes, and litter success post release. Furthermore, improvement in immune function could be investigated through safe immune challenge bioassays [211].

Metapopulation management has been proposed for a number of canids that are endangered or are limited to small spatially fragmented landscapes, including the red wolf, European grey wolf, and Ethiopian wolf [27,28,212]. Appeasing pheromones could improve the efficacy and welfare of the conservation management of these species. Moreover, such pheromones could help mitigate aggression toward humans in problem populations of other wild canids, such as Australian dingoes, during breeding and whelping seasons [213]. They could also help improve captive breeding and reintroduction programs for species such as the Arctic fox in Norway [22]. Given the species-specific nature of pheromones [154] and the possibility of an increased effectiveness when derived from and used upon the target species, it is possible that appeasing pheromones isolated from each species of canid may elicit a greater reduction in stress- and aggression-related hormones and behaviours than using a cross-species dog appeasing pheromone. This would offer a natural, non-invasive tool to modify behaviour in a number of wild canids. This is particularly important to improve the success of translocation and pack formation in species, such as endangered wolves, foxes, and even dingoes, that require metapopulation management in the face of habitat fragmentation, disease, and human–wildlife conflict.

## 6. Conclusions

The management of wild canids is necessary to ensure the continued survival of many species due to habitat fragmentation and human persecution. This is most successfully achieved through metapopulation management, where artificially bonded packs/pairs of animals are held in acclimatisation pens for a period of time prior to release. However, temporary captivity leads to chronic stress and possible aggression, which may reduce fitness through suppressed immunity and/or reproduction, resulting in poor translocation outcomes. Current captive methods of castration and pharmaceutical sedation to manage stress and aggression are inadvisable and counterproductive for use in wild canids. Pheromones are naturally occurring chemical messages transmitted between conspecifics that act on the endocrine system to modulate behaviours. Most animals can differentiate these highly species-specific pheromones between conspecific and heterospecific individuals based on small molecular differences in the pheromone composition. Species-specific appeasing pheromones have been shown to reduce stress and aggression behaviours in domestic animals. Previous research on African wild dogs suggests that domestic dog appeasing pheromones can work cross-species to reduce aggression in established captive packs after stressful intervention through a reduction in both contact dominance and testosterone surges. Further research is required to investigate whether a domestic dog or a species-specific appeasing pheromone is able to directly reduce glucocorticoid levels and stress. This can be achieved through an integrative approach using bioassays for behavioural stress (ethogram) and cortisol (non-invasive faecal hormonal assays) in animals exposed to changing synthetic appeasing analogues. This tool has the potential to significantly increase the welfare of animals during conservation intervention. Reduced stress and aggression may lead to tangible translocation and artificial pack formation results. For example, managers should be able to observe reduced aggression between unfamiliar animals; accelerated and increased rates of successful pack bonding, thereby reducing the time spent in captivity prior to release; as well as an elevated immune function that could manifest in (i) reduced disease-induced mortality shortly after release and (ii) increased breeding success and first-litter pup survival.

## Figures and Tables

**Table 1:** Comparison of the number of the major histocompatibility complex class II, DLA-DQA1, and DLA-DQB1 (dog leukocyte antigen-DQ α1 and β1 respectively) alleles found in different canid populations [8,11,12,13,14,15].

Study Species	DLA-DQA1	DLA-DQB1
Domestic Dog: European purebred dogs *n* = > 8000	18	47
African wild dog: Eastern and Southern Africa *n* = 368	1	2
Grey wolf: Canada and Alaska *n* = 194	12	15
Grey Wolf: Northern Europe *n* = 163	9	10
Grey wolf: Total *n* = 407	18	21
Mexican wolf: Captive American Population *n* < 7	5	3
Ethiopian wolf: Bale Mountains Ethiopia *n* = 99	2	5

**Table 2:** Comparison of different fatty acid ratios that constitute the appeasing pheromone identified for different mammals [199].

Component	Canine % (*w*/*w*)	Porcine % (*w*/*w*)	Caprine % (*w*/*w*)	Bovine % (*w*/*w*)	Ovine % (*w*/*w*)	Equine % (*w*/*w*)
Oleic acid	21.5–27.8	24.7–36.8	20.1–22.3	24.9–28.6	32.8–38.8	35.2–40.3
Palmitic acid	20.8–24.9	15.5–26.8	22.3–26.8	19.2–23.1	21.6–25.9	22.8–26.7
Linoleic acid	20.5–25.4	29.5–40.6	20.2–22.5	20.5–24.3	21.2–25.7	22.1–27.1
Myristic acid	2.2–3.9	3.9–9.6	8.5–10.1	3.2–5.6	3.4–5.9	2.0–2.8
Lauric acid	0.4–1.8	2.8–8.7	11.4–14.8	1.9–4.2	2.6–4.4	2.3–3.7
Pentadecanoic acid	1.8–3.1					
Cholesterol	10.2–18.6					
Capric acid		0.5–3.5				
Squalene			9.5–11.2			
1-docosanol 2,2-dimethyl 1,3-dioxolane 4-methanol				18.4–22.8	7.4–9.7	4.4–6.7

*w*/*w* = weight for weight.

## Data Availability

The data presented in this study are available on request from the corresponding author.
